# Improved Cylinder-Based Tree Trunk Detection in LiDAR Point Clouds for Forestry Applications

**DOI:** 10.3390/s25030714

**Published:** 2025-01-24

**Authors:** Shaobo Ma, Yongkang Chen, Zhefan Li, Junlin Chen, Xiaolan Zhong

**Affiliations:** College of Resources and Environment, South China Agricultural University, Guangzhou 510642, China; msb2021@stu.scau.edu.cn (S.M.); lizhefan@stu.scau.edu.cn (Z.L.); chenjl@scau.edu.cn (J.C.)

**Keywords:** individual tree trunk detection, terrestrial LiDAR, random sample consensus cylinder fitting, point cloud

## Abstract

The application of LiDAR technology in extracting individual trees and stand parameters plays a crucial role in forest surveys. Accurate identification of individual tree trunks is a critical foundation for subsequent parameter extraction. For LiDAR-acquired forest point cloud data, existing two-dimensional (2D) plane-based algorithms for tree trunk detection often suffer from spatial information loss, resulting in reduced accuracy, particularly for tilted trees. While cylinder fitting algorithms provide a three-dimensional (3D) solution for trunk detection, their performance in complex forest environments remains limited due to sensitivity to parameters like distance thresholds. To address these challenges, this study proposes an improved individual tree trunk detection algorithm, Random Sample Consensus Cylinder Fitting (RANSAC-CyF), specifically optimized for detecting cylindrical tree trunks. Validated in three forest plots with varying complexities in Tianhe District, Guangzhou, the algorithm demonstrated significant advantages in the inlier rate, detection success rate, and robustness for tilted trees. The study showed the following results: (1) The average difference between the inlier rates of tree trunks and non-tree points for the three sample plots using RANSAC-CyF were 0.59, 0.63, and 0.52, respectively, which were significantly higher than those using the Least Squares Circle Fitting (LSCF) algorithm and the Random Sample Consensus Circle Fitting (RANSAC-CF) algorithm (*p* < 0.05). (2) RANSAC-CyF required only 2 and 8 clusters to achieve a 100% detection success rate in Plot 1 and Plot 2, while the other algorithms needed 26 and 40 clusters. (3) The effective distance threshold range of RANSAC-CyF was more than twice that of the comparison algorithms, maintaining stable inlier rates above 0.9 across all tilt angles. (4) The RANSAC-CyF algorithm still achieved good detection performance in the challenging Plot 3. Together, the other two algorithms failed to detect. The findings highlight the RANSAC-CyF algorithm’s superior accuracy, robustness, and adaptability in complex forest environments, significantly improving the efficiency and precision of individual tree trunk detection for forestry surveys and ecological research.

## 1. Introduction

Forests are vital to the global ecosystem, playing a key role in biodiversity conservation and environmental regulation [1]. Single tree identification, a critical technique for locating and classifying individual trees, serves as a fundamental component in forest resource monitoring. It provides essential data for analyzing forest structure, monitoring dynamic changes, and evaluating ecosystem services, enabling efficient and accurate acquisition of tree-level information.

In recent years, 3D laser scanning technology has made remarkable development and application in forestry and ecology. This technology uses laser radar equipment to accurately retrieve parameters such as trunk, crown shape, tree height, crown diameter, and leaf area index without any contact. The traditional forestry investigation method has some limitations in efficiency and accuracy, and the introduction of 3D laser scanning technology has significantly improved the speed and accuracy of data collection, providing a new solution for forest resource monitoring and ecological environment assessment [2]. Especially for single tree Diameter at Breast Height (DBH) extraction and acceptable tree measurement, 3D laser scanning technology shows unique advantages, providing a more reliable tool for accurate forest management and ecosystem service assessment.

Single tree identification is crucial to realize accurate extraction of DBH of trees [3]. Based on the segmentation and feature extraction of LiDAR point cloud data, scholars at home and abroad have carried out much related research, among which the commonly used algorithms mainly include an algorithm based on 2D circle fitting [4,5,6,7,8,9,10,11] and an algorithm based on cylinder fitting [12,13,14,15,16]. Two-dimensional circle fitting algorithms, such as Least Squares Circle Fitting, (LSCF) and Random Sample Consensus Circle Fitting (RANSAC-CF), are widely used because of their simple calculation and high efficiency. For example, Sheng [17] and others proposed a circle-fitting algorithm based on Intensity Weighted Least Squares (IWLS), which can effectively improve the extraction accuracy of forest DBH of moving laser scanning point cloud data and reduce the influence of poorly aligned point clouds on DBH fitting. However, this kind of algorithm needs to project the 3D point cloud data on the two-dimensional plane, which leads to the loss of some spatial information and then affects the accuracy of single tree recognition [18,19]. In addition, when the trees are inclined, data redundancy and model errors readily occur in the process of 2D projection, which leads to a decline in recognition accuracy.

In contrast, the cylindrical fitting algorithm can directly process the point cloud data in 3D space, which can preserve the spatial structure information of the trunk more completely and effectively solves the problem of tree inclination. For example, Zhang [20] and others put forward a solid-state LiDAR inertial ranging method to enhance the semantics in the dense tree environment and used the adaptive segmented cylinder fitting method to account for curved trunks, thus improving the positioning accuracy. However, in the case of complex woodland scenes or low accuracy of point cloud data, the robustness and recognition success rate of the cylinder fitting algorithm may be limited, and it also shows high sensitivity to the setting of parameters (such as distance threshold) [21,22].

In view of the strong robustness, flexible model selection, wide application range, and high computational efficiency of the RANSAC algorithm [23,24,25], this study proposes a single tree identification algorithm based on Random Sample Consensus Cylinder Fitting (RANSAC-CyF) and designs an effective iterative optimization method to determine and optimize the algorithm parameters. This method carries out cylinder fitting in three-dimensional space, makes full use of the spatial information of point cloud data, realizes the efficient and stable identification of a single tree through iterative optimization of distance threshold and interior point rate threshold, and has good environmental adaptability.

## 2. Materials and Methods

### 2.1. Materials

#### 2.1.1. Study Area

**Figure 1:**
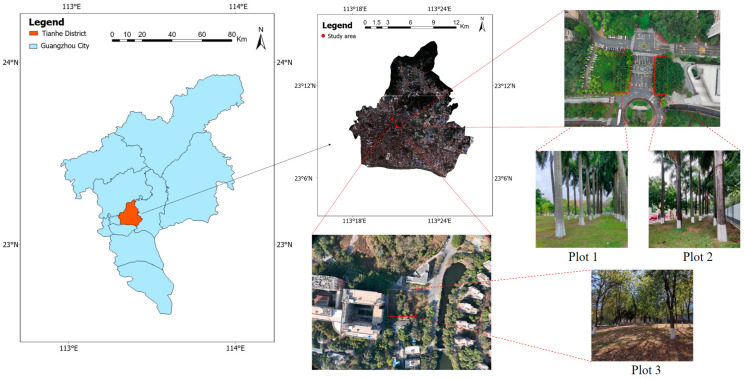
Schematic map of the study area.

The study area is located in the plantation forest land (23°9′36″ N, 113°13′48″ E) of South China Agricultural University in Guangzhou, Guangdong Province, China. As shown in Figure 1, Guangzhou is located in south China and belongs to the subtropical monsoon climate zone. Summer is hot and rainy, winter is relatively warm, and the transition between spring and autumn is mild. The annual average temperature is between 21.7 and 23.1 degrees Celsius, and the highest is 39.1 degrees Celsius. The annual precipitation is 193 mm, and the average annual precipitation days are 149 days.

Three plots with varying environmental complexities at South China Agricultural University in Guangzhou were selected for the study. Plot 1 contains 27 trees and covers an area of approximately 50 m × 20 m. It is characterized by an open area with minimal interference and a single tree species (Royal Palm trees) with an average Diameter at Breast Height (DBH) of 0.491 ± 0.041 m. The trees in Plot 1 are arranged neatly without debris interference, making it an ideal forest environment for evaluating the performance of individual tree detection under controlled conditions. Plot 2 contains 25 trees and covers an area of approximately 40 m × 15 m. The average DBH of trees in this plot is similar to that of Plot 1, but the environmental conditions are relatively more complex, including the presence of a stone archway, walls, shrubs, and other features that may interfere with individual tree detection.

To address concerns about the limited diversity of study plots and tree species, a third plot (Plot 3) was added to the study. Plot 3 is located approximately 1 km northwest of Plots 1 and 2, near an experimental building within the forested area of South China Agricultural University. It covers an area of approximately 30 m × 30 m and is distinct from Plots 1 and 2 in terms of both environmental characteristics and tree composition. Plot 3 features a random distribution of smaller trees, primarily Bauhinia, with relatively thin trunks and no fixed planting arrangement. The average DBH of the trees in Plot 3 is 0.248 ± 0.036 m, with the smallest DBH measuring just 0.188 m and the largest DBH reaching 0.400 m.

By comparing the results from all three plots, the study provides a comprehensive evaluation of the proposed algorithm’s robustness and adaptability under varying environmental complexities and tree species. The inclusion of Plot 3 not only enhances the representativeness of the study but also addresses potential limitations in generalizing the algorithm to forests with higher variability in tree size and arrangement.

#### 2.1.2. Data Acquisition and Processing

The experimental data were acquired using a STONEX X150 plus terrestrial 3D laser scanner, manufactured by STONEX, located in Paderno Dugnano, Milan, Italy. This device supports a distance measurement range of 2–150 m and a high-speed scanning capability of over 40,000 points per second, featuring a 360° horizontal and 180° vertical panoramic scanning field of view, 0.37 milliradian high angular resolution, and high-precision point cloud acquisition performance of ±4 mm at 50 m.

**Figure 2:**
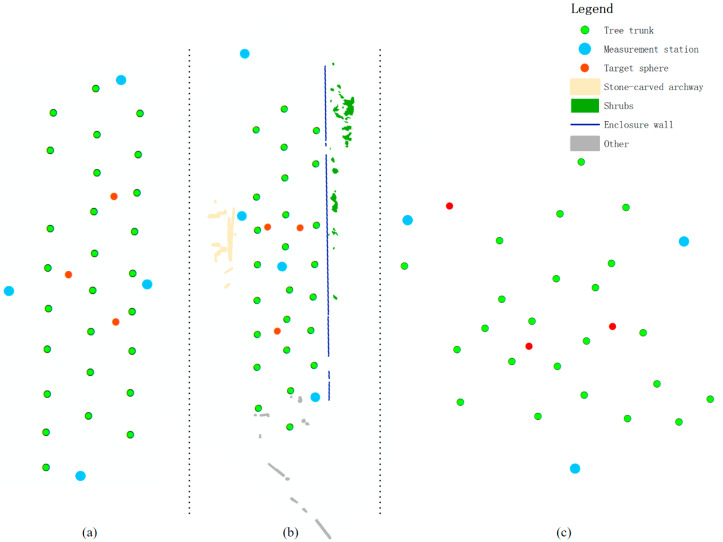
Element distribution maps for Plot 1 (**a**), Plot 2 (**b**), and Plot 3 (**c**).

In all three plots, survey stations and target balls were set up to facilitate data collection [26]. Specifically, four survey stations and three target balls were used in Plots 1 and 2, while three survey stations and three target balls were used in Plot 3. The stations were distributed throughout the plot, ensuring that each station had a direct line of sight to all the target balls (Figure 2).

**Figure 3:**
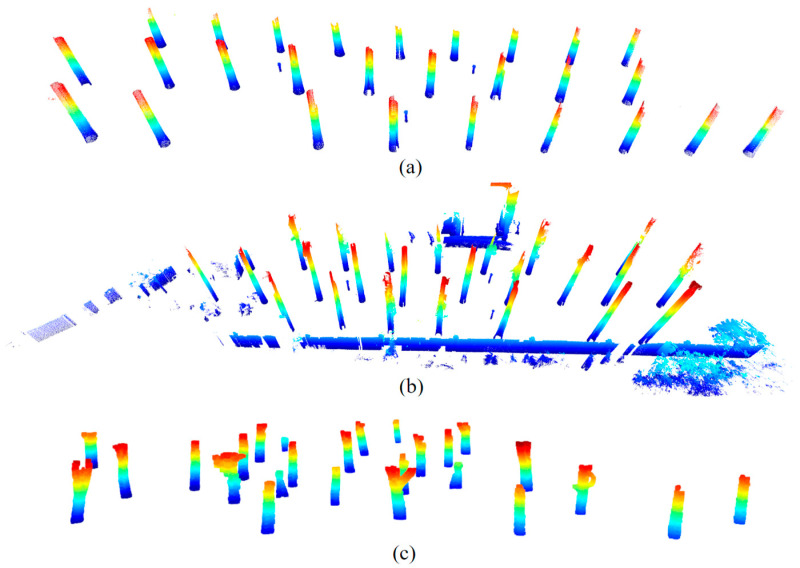
Visualization of preprocessed point cloud data, where colors are assigned based on height values, for (**a**) Plot 1, (**b**) Plot 2, and (**c**) Plot 3.

After completing outdoor data collection, initial preprocessing, such as trimming, stitching, and data registration, was performed using StoNexSiScan [27]. However, the point cloud data were extensive, exceeding 30 million points. Therefore, the stitched point cloud was downsampled by 20% [28] to improve rendering and computation efficiency. Next, elevation normalization was carried out to eliminate the effects of terrain. Finally, the ground and foliage were cropped to observe the forest elements more clearly, resulting in the preprocessed data (Figure 3).

#### 2.1.3. Experimental Environment and Parameter Settings

This experiment used Python 3.6, based on open-source libraries such as open3d and PCL. The computer running the experiments had a CPU model of 13th Gen Intel (R) Core (TM) i9-13900K and 32 GB of RAM.

After data preprocessing, the point cloud data were sliced toward height. Considering the similarity between the shape of the tree DBH and a cylinder, the slicing operation was performed at the DBH position. The chosen slice thickness for the experiment was 20 cm, corresponding to the DBH region from 1.2 to 1.4 m above the ground. After slicing, the slice data were processed using clustering analysis methods, followed by individual tree detection analysis on the point cloud clusters obtained from each cluster.

During cylinder fitting, the number of neighboring points *k* for normal calculation was set to 50 to ensure accuracy while avoiding excessive computational load. The normal distance *weight* was set to 0.05, a value determined based on a comprehensive analysis of existing research and empirical testing [29]. Given that the accuracy of the point cloud data in this experiment is approximately 0.03 m, the initial value for the distance threshold *t* was set to 0.03 m, with an adjustment step ∆*t* of 0.005 m. Considering the DBH variation of individual trees across the three plots, the acceptable range for the cylinder model diameter was set from 0 to 0.8 m during individual tree detection to ensure fitting accuracy and effectiveness.

### 2.2. Methods

#### 2.2.1. Principle of Cylinder Fitting and Cylinder Model

Considering that the cross-section of a tree’s main trunk is generally circular and extends upward in the form of a cylinder, and the laser emitted by a terrestrial LiDAR scanner only reaches the tree bark and immediately returns (occasionally penetrating part of the bark, but the penetration depth is negligible) [30], the normal DBH point cloud slice should be an approximately hollow cylinder. By utilizing this feature, we can use the cylinder model to distinguish the trunk point cloud clusters effectively.

The expression of the cylinder model is

(1) $${\left(x-x_{0}\right)}^{2}+{\left(y-y_{0}\right)}^{2}+{\left(z-z_{0}\right)}^{2}-[\alpha (x-x_{0})+\beta (y-y_{0})+\gamma (z-z_{0})]=r^{2}$$

**Figure 4:**
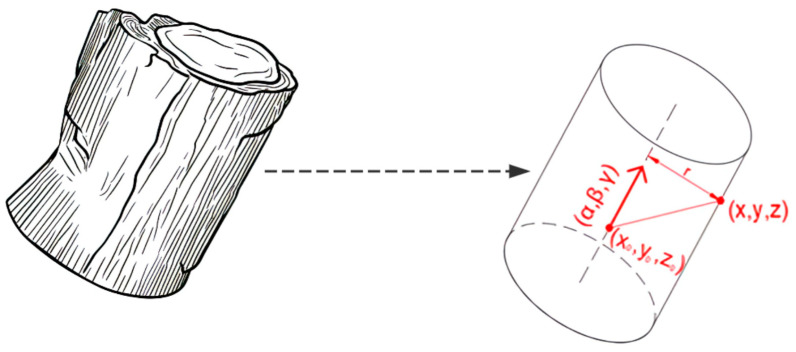
Cylindrical model expression.

In Equation (1), $\left(x_{0},y_{0},z_{0}\right)$ represents a reference point on the cylinder axis, $\left(\alpha ,\beta ,\gamma \right)$ denotes the unit direction vector of the cylinder axis, and r is the radius of the cylinder, as shown in Figure 4.

#### 2.2.2. Cylinder Model Calculation

To perform cylinder fitting, it is necessary to first calculate the normals for each point in the input point cloud. The principal steps [31] are as follows:

(1) For each point $p_{i}$ , use k-d trees [32] to search for the k nearest neighbor points ${\left\{p_{i}^{j}\right\}}_{j=1}^{k}$ within a specified search radius.
(2) Using the set of neighbor points ${\left\{p_{i}^{j}\right\}}_{j=1}^{k}$ for $p_{i}$, calculate the covariance matrix $C_{i}$:
  (2) $$C_{i}=\frac{1}{k}\sum_{j=1}^{k}\left(p_{i}^{j}-{\bar{p}}_{i}\right){\left(p_{i}^{j}-{\bar{p}}_{i}\right)}^{T}$$
  In Equation (2), ${\bar{p}}_{i}$ is the mean of the set of neighbor points.
  (3) $${\bar{p}}_{i}=\frac{1}{k}\sum_{j=1}^{k}p_{i}^{j}$$
(3) Perform eigenvalue decomposition on the covariance matrix $C_{i}$ to obtain the eigenvector $n_{i}$ corresponding to the smallest eigenvalue $\lambda_{min}$. This eigenvector is the normal direction of point $p_{i}$:
  (4) $$C_{i}n_{i}=\lambda_{min}n_{i}$$
(4) Store the calculated normal $n_{i}$ in the data structure of the output point cloud.
(5) Repeat the above process for all points to obtain the normals for the entire point cloud ultimately.

After determining the normals of the point cloud, any two points and their normals from the point cloud are selected to calculate the fitting cylinder model. The principal steps [33] are as follows:

(1) Arbitrarily select two points $P_{1}=(x_{1},y_{1},z_{1})$ and $P_{2}=(x_{2},y_{2},z_{2})$ from the point cloud, with the corresponding unit normal vectors $N_{1}=(n_{1x},n_{1y},n_{1z})$ and $N_{2}=(n_{2x},n_{2y},n_{2z})$.
(2) Construct two parametric lines based on $P_{1}$, $P_{2}$, $N_{1}$, and $N_{2}$:
  (5) $$L_{1}:P_{1}+tN_{1}=\left(\begin{array}{c} x_{1}\\ y_{1}\\ z_{1} \end{array}\right)+t\left(\begin{array}{c} n_{1x}\\ n_{1y}\\ n_{1z} \end{array}\right)$$
  (6) $$L_{2}:P_{2}+tN_{2}=\left(\begin{array}{c} x_{2}\\ y_{2}\\ z_{2} \end{array}\right)+t\left(\begin{array}{c} n_{2x}\\ n_{2y}\\ n_{2z} \end{array}\right)$$

In Equations (5) and (6), t represents the parameter along the direction of the normal line. Using the least squares method to calculate the parameters $t_{1}$ and $t_{2}$, the closest points on $L_{1}$ and $L_{2}$ are $Q_{1}$ and $Q_{2}$, respectively, can be expressed as:

(7) $$Q_{1}=P_{1}+t_{1}N_{1}$$

(8) $$Q_{2}=P_{2}+t_{2}N_{2}$$

The vector $Q_{1}Q_{2}$ gives the direction vector D of the cylinder axis. After normalization, the unit vector D is obtained:

(9) $$D=\frac{Q_{1}Q_{2}}{\left\|Q_{1}Q_{2}\right\|}$$

(3) The reference point on the cylinder axis is chosen as the midpoint C of $Q_{1}$ and $Q_{2}$:
  (10) $$C=\frac{Q_{1}+Q_{2}}{2}$$
(4) Calculate the distance r from $P_{1}$ to the cylinder axis as the radius.
(5) Summarize the cylinder model parameters, including the reference point coordinates on the cylinder axis (Cx,Cy,Cz), the unit direction vector of the axis (Dx,Dy,Dz), and the cylinder radius r.

#### 2.2.3. Inlier Determination

An inlier is a point in the fitted model with an error smaller than a predetermined threshold. In the fitting process of RANSAC-CyF, inlier determination is one of the algorithm’s core steps.

For a point A in the point cloud set, with its unit normal vector N, the steps to determine whether it is an inlier of the cylinder model are as follows:

(1) Set the distance threshold t and the weight for the normal vector distance weight.
(2) Calculate the absolute value d of the difference between the distance from A to the cylinder axis and the cylinder model radius.
(3) Calculate the projection $A^{\prime }$ of A on the cylinder axis, and then calculate the angle θ between $A^{\prime }$ and N:
  (11) $$\theta =\arccos\left(\frac{AA^{\prime }\cdot N}{\left\|AA^{\prime }\right\|\cdot \left\|N\right\|}\right)$$
(4) Calculate the total distance distance obtained by weighting d and θ.
  (12) $$distance=d\times \left(1-weight\right)+\theta \times weight$$

**Figure 5:**
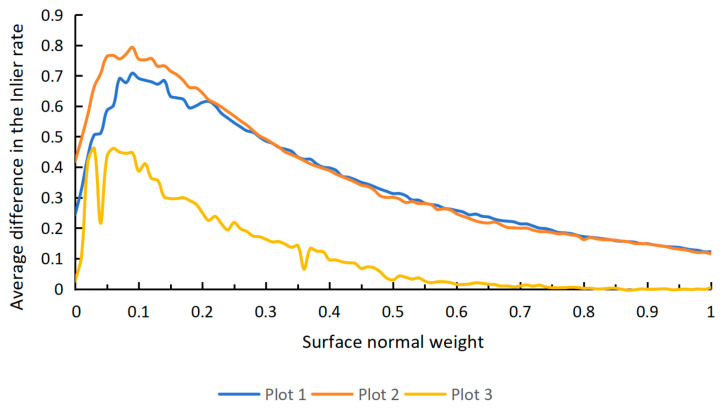
Distribution of the average inlier rate differences between trunk and non-trunk point clouds with varying surface normal weights.

In Equation (12), weight is used to adjust the relative importance of the angle difference between $AA^{\prime }$ and N and the absolute value d when calculating distance, where $weight\in \left[0\right.,\left.1\right]$. A lower weight emphasizes the geometric distance, while a higher weight gives more importance to the angle deviation. To determine a reasonable surface normal weight, this study analyzed the distribution of differences in the average inlier rate between trunk point clouds and non-trunk point clouds across three sample plots with varying surface normal weights. Considering that the point cloud alignment precision in this study is approximately 0.03 m, the distance threshold was set to 0.03 m. The results indicate that when the surface normal weight is set between 0.05 and 0.1, the inlier rate differences reach their maximum across all three sample plots, making 0.05 a suitable weight value. Furthermore, Figure 5 shows that the weight setting has a high tolerance, meaning that accurate cylinder fitting can still be achieved without selecting the precise weight value that maximizes the inlier rate difference.

(5) Compare distance with t. If distance is less than or equal to t, A is considered an inlier; otherwise, A is considered an outlier.

#### 2.2.4. Cylinder Fitting

**Figure 6:**
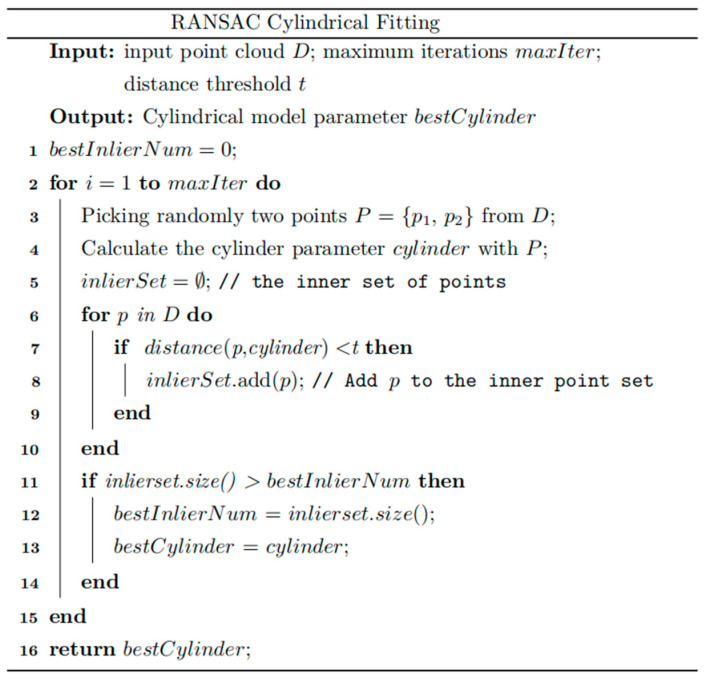
Cylindrical fitting pseudo-code.

Cylinder fitting based on RANSAC (Random Sample Consensus) analyzes the point cloud and uses an iterative process to optimize and automatically identify the best-fitting cylinder model. Figure 6 shows the cylindrical fitting pseudo-code.

The algorithm’s input parameters are the point cloud set D, the maximum number of iterations maxIter, and the distance threshold t. Its output is the parameters of the best-fitted cylinder model (see Section 2.2.1). The algorithm’s time complexity is $O\left(k\times n\right)$, where k is the maximum number of iterations and n is the total number of points in the point cloud. The algorithm’s space complexity is $O\left(n\right)$.

#### 2.2.5. Individual Tree Detection

The inlier rate refers to the ratio of the number of inliers to the total number of data points in the RANSAC-CyF algorithm. A higher inlier rate indicates fewer points are classified as noise or outliers, and the fitted model explains the dataset more effectively [34]. Since the shape of the trunk point cloud is approximately cylindrical, the inlier rate of the fitted cylinder model is much higher for trunk point clouds than for non-trunk point clouds. Therefore, individual tree detection can be performed based on the inlier rate. RANSAC-CyF sets an inlier rate threshold (depending on the minimum inlier rate of trunk points and the maximum inlier rate of non-trunk points) as the criterion for individual tree detection [35]. If the inlier rate of a fitting exceeds the inlier rate threshold and the diameter of the fitted cylinder is within the standard error range of the actual expected DBH size, the detection is considered successful.

**Figure 7:**
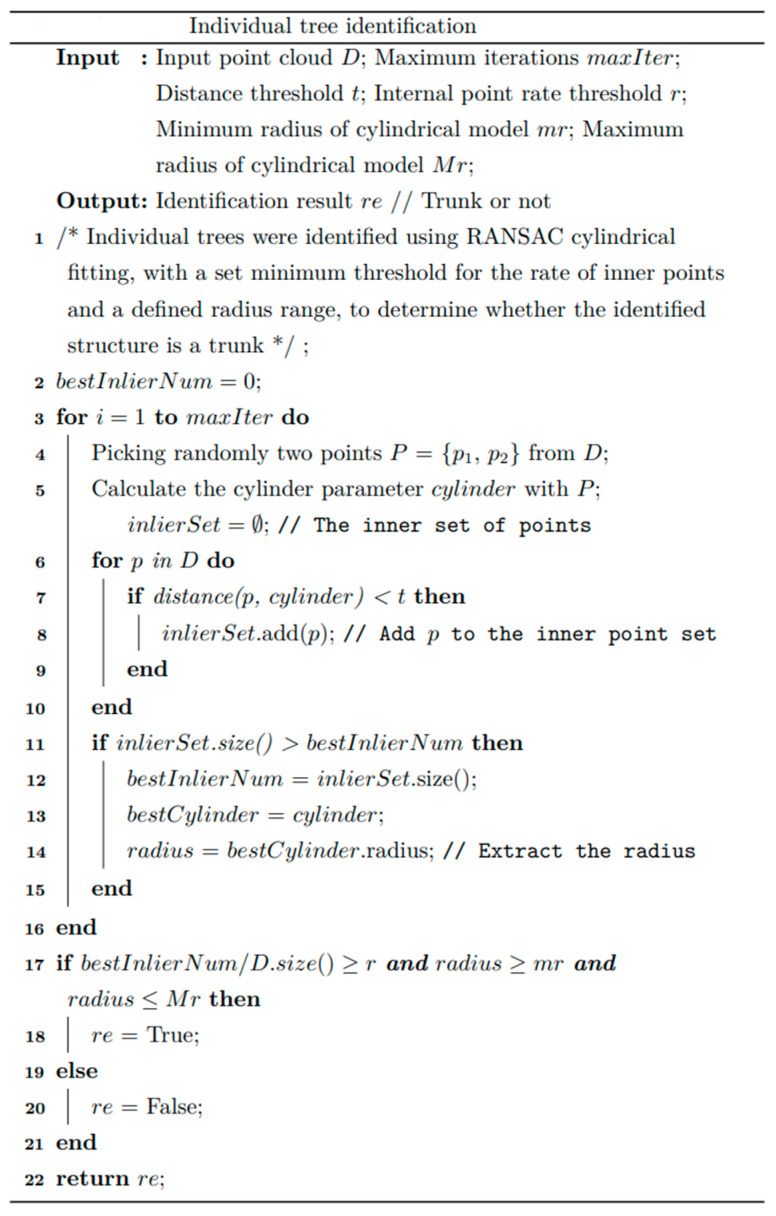
Individual tree identification pseudo-code.

Figure 7 shows the individual tree trunk identification pseudo-code. The algorithm’s input parameters are the point cloud set D, the maximum number of iterations maxIter, the distance threshold t, and the inlier rate threshold r. The output parameter is a Boolean value representing the result of individual tree detection. The algorithm’s time complexity is $O\left(k\times n\right)$, where k is the maximum number of iterations and n is the total number of points in the point cloud. The algorithm’s space complexity is $O\left(n\right)$.

The inlier rate depends on multiple factors, including the accuracy of the point cloud data and the setting of the distance threshold, among others [36]. The actual environmental conditions determine the settings for the distance threshold and the inlier rate threshold. During the individual tree detection process, if there are many false positives where non-trunk point clouds are incorrectly identified as trunk point clouds, it indicates that the inlier rate threshold is too low and should be appropriately raised to reduce false positives. Conversely, if there are frequent false negatives where some suitable trunk point clouds are not identified, it indicates that the threshold is too high and should be appropriately lowered.

The steps for setting the distance threshold t and the inlier rate threshold r are as follows:

(1) Extract a small number of volumes from the total clustered samples to form a set M, and classify the volumes in M into tree trunk samples $M_{1}$ and non-trunk samples $M_{2}$, such that $M=M_{1}\cup M_{2}$ and $M_{1},M_{2}\neq O$. Based on the accuracy of the point cloud data, initially set the distance threshold t. Generally, more accurate point cloud data can have a smaller initial distance threshold.
(2) Perform cylinder fitting on each volume in $M_{1}$ and $M_{2}$, and respectively calculate the minimum inlier rate $a_{1}$ for the volumes in $M_{1}$ and the maximum inlier rate $a_{2}$ for $M_{2}$.
(3) Adjust the distance threshold t (i.e., t=±Δt) and repeat step (2).
(4) Repeat step (3) until the difference $\left(a_{1}-a_{2}\right)$ is maximized, then terminate the loop and retain the distance threshold t at that point.
(5) If $a_{1}$ is greater than $a_{2}$, the initial setting for the inlier rate threshold r is:
  (13) $$r=\frac{a_{1}+a_{2}}{2}$$
  If $a_{1}\leq a_{2}$, the algorithm cannot ensure good detection results for the given data.

## 3. Results

According to the clustering results of the point cloud data from the three plots, the total number of clusters in both plots exceeded the number of trunk point cloud clusters (Table 1), especially in Plot 2, where the total number of clusters was more than twice the number of trunk point cloud clusters. Therefore, further individual tree detection analysis was required to determine which clusters represented individual trees.

This study comprehensively evaluated the RANSAC-CyF algorithm’s performance in individual tree detection by comparing its fitting results with those of the commonly used LSCF and RANSAC-CF algorithms. We compared the algorithms’ tree trunk detection performance in four aspects: inlier fitting rate, detection success rate, robustness, and fitting of tilted trees.

### 3.1. Inlier Rate of Fitting

**Figure 8:**
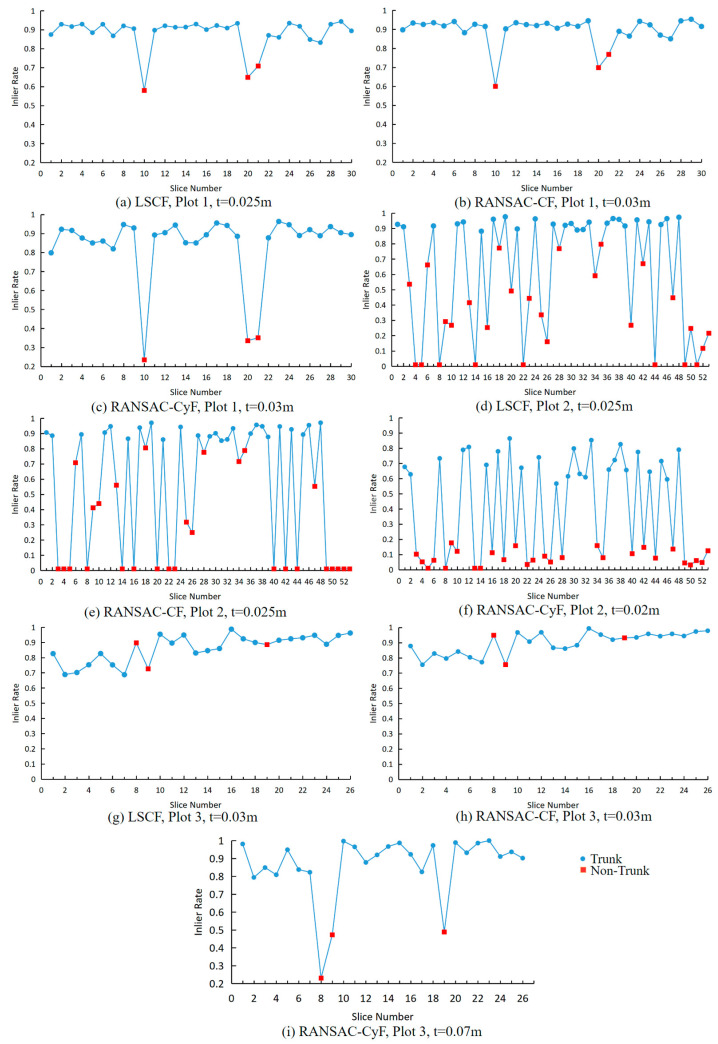
Fitting results for the three algorithms: (**a**) LSCF, Plot 1; (**b**) RANSAC-CF, Plot 1; (**c**) RANSAC-CF, Plot 1; (**d**) LSCF, Plot 2; (**e**) RANSAC-CF, Plot 2; (**f**) RANSAC-CF, Plot 2; (**g**) LSCF, Plot 3; (**h**) RANSAC-CF, Plot 3; (**i**) RANSAC-CF, Plot 3.

Figure 8 shows the fitting results of the three algorithms under their respective optimal distance thresholds. The optimal distance thresholds for each algorithm in different plots were calculated using the method described in Section 2.2.5. However, in Plot 3, the LSCF and RANSAC-CF algorithms failed to achieve appropriate distance thresholds (as *a*_1_ was always smaller than *a*_2_, as explained in Section 2.2.5), indicating poor fitting performance. Therefore, the LSCF and RANSAC-CF algorithms did not have optimal distance thresholds in Plot 3. To demonstrate their fitting results, a fixed distance threshold of 0.03 m was used for both algorithms in this plot.

In Plot 1, all three algorithms exhibited high and stable inlier rates for trunk point clouds, with the average inlier rates of LSCF, RANSAC-CF, and RANSAC-CyF algorithms being 0.9, 0.92, and 0.9, respectively. However, for non-trunk point clouds, the average inlier rate of the RANSAC-CyF algorithm was 0.31, which was significantly lower than that of the LSCF algorithm (0.65) and the RANSAC-CF algorithm (0.69) (*p* < 0.05).

In Plot 2, although the LSCF and RANSAC-CF algorithms achieved average inlier rates above 0.9 for trunk point clouds, these two algorithms exhibited poor stability in inlier rates for non-trunk point clouds, with average inlier rates of 0.32 ± 0.27 and 0.23 ± 0.31, respectively, both having a range of 0.79, with some non-trunk point clouds having inlier rates close to 0.8. In contrast, the RANSAC-CyF algorithm showed a significant difference between the inlier rates for trunk and non-trunk point clouds, with average inlier rates of 0.71 ± 0.09 and 0.08 ± 0.05, respectively, with the inlier rates for non-trunk point clouds all being less than 0.2.

In Plot 3, the average inlier rates for non-trunk point clouds using the LSCF and RANSAC-CF algorithms were 0.84 and 0.88, respectively, while the average inlier rates for trunk point clouds were 0.86 and 0.9, respectively, making it impossible to distinguish between trunk and non-trunk point clouds. In contrast, the RANSAC-CyF algorithm achieved inlier rates exceeding 0.8 for trunk point clouds, while the inlier rates for non-trunk point clouds were all below 0.5, with the non-trunk inlier rates remaining significantly lower than those of trunk point clouds.

To distinguish between trunk and non-trunk point clouds more efficiently, we introduced the concept of “discrimination value”. This value, defined as the difference between the minimum inlier rate for trunk point clouds and the maximum inlier rate for non-trunk point clouds, was used to evaluate the algorithm’s performance. When the discrimination value exceeds 0, a suitable inlier rate threshold can be set to achieve perfect individual tree detection (i.e., 100% detection rate with 0% false positives). The size of the discrimination value directly reflects the range of inlier rate thresholds for perfect individual tree detection. The larger the discrimination value, the greater the flexibility in choosing the inlier rate threshold, demonstrating the algorithm’s robustness.

As shown in Table 2, the discrimination values of the RANSAC-CyF algorithm in Plot 1 and Plot 2 were 0.447 and 0.390, respectively, significantly higher than those of the LSCF and RANSAC-CF algorithms (*p* < 0.05). In Plot 3, the LSCF and RANSAC-CF algorithms were unable to produce valid discrimination values, indicating poor performance in distinguishing between trunk and non-trunk point clouds under the given conditions. In contrast, the RANSAC-CyF algorithm achieved a discrimination value of 0.305, demonstrating its robustness and effectiveness in handling complex environments with small, randomly distributed trees.

### 3.2. Number of Samples and Detection Success Rate

In practical individual tree detection, since it is not possible to predetermine whether a cluster is a trunk cluster or a non-trunk cluster, the inlier rate threshold should be set using the sampling method described in Section 2.2.5 for individual tree detection. Therefore, this study evaluated the performance of the algorithms in practice by analyzing the success rate in achieving perfect individual tree detection (i.e., 100% detection rate with 0% false positives) under different sampling numbers. If a high perfect individual tree detection rate can be achieved with fewer samples, the algorithm has good individual tree detection performance and practical applicability.

Based on the method described in Section 2.2.5 for setting the inlier rate threshold, this study analyzed the relationship between the success rate in achieving perfect individual tree detection and the number of samples for the three algorithms. The specific method was as follows: Fit and distinguish the randomly sampled point clouds, divide them into trunk point clouds and non-trunk point clouds, and calculate the minimum inlier rate $a_{1}$ of the trunk point clouds and the maximum inlier rate $a_{2}$ of the non-trunk point clouds. If there are no trunk point clouds in the sample, $a_{1}$ is set to 1.0; if there are no non-trunk point clouds in the sample, $a_{2}$ is set to 0. The inlier rate threshold is set as the average of $a_{1}$ and $a_{2}$. For each number of samples, the success rate in achieving perfect individual tree detection is obtained by iterating 10^5^ times.

**Figure 9:**
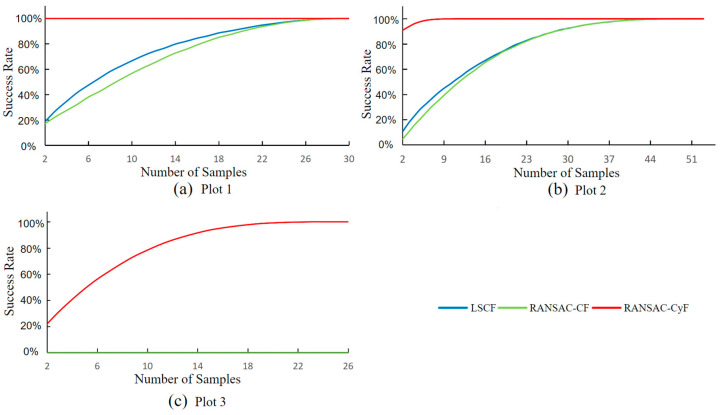
Distribution of the success rates in achieving perfect individual tree detection with different numbers of samples for the three algorithms: (**a**) Plot 1; (**b**) Plot 2; (**c**) Plot 3.

As shown in Figure 9, the RANSAC-CyF algorithm achieved a 100% perfect individual tree detection success rate in Plot 1 with only two randomly selected clusters. In Plot 2, the algorithm achieved a 91% success rate with two samples and reached 100% with more than eight samples. In Plot 3, the performance of the RANSAC-CyF algorithm slightly declined, achieving over 80% success rate with 10 samples. In contrast, the LSCF and RANSAC-CF algorithms had significantly lower success rates. In Plots 1 and 2, their success rates were below 20% with 2 samples and only reached 100% when the number of samples increased to 26 (Plot 1) and 40 (Plot 2). In Plot 3, neither the LSCF nor the RANSAC-CF algorithm achieved perfect individual tree detection.

### 3.3. Robustness Analysis

Determining an appropriate distance threshold is crucial in individual tree detection. However, in practical applications, the initial setting and optimal value of the distance threshold often have unstable deviations, and the adjustment step size ∆t cannot be infinitely small, making it unrealistic to determine the optimal distance threshold precisely. Therefore, a good algorithm should exhibit strong robustness when selecting the distance threshold. This study analyzed the robustness of the three algorithms by examining the discrimination values under different distance thresholds.

**Figure 10:**
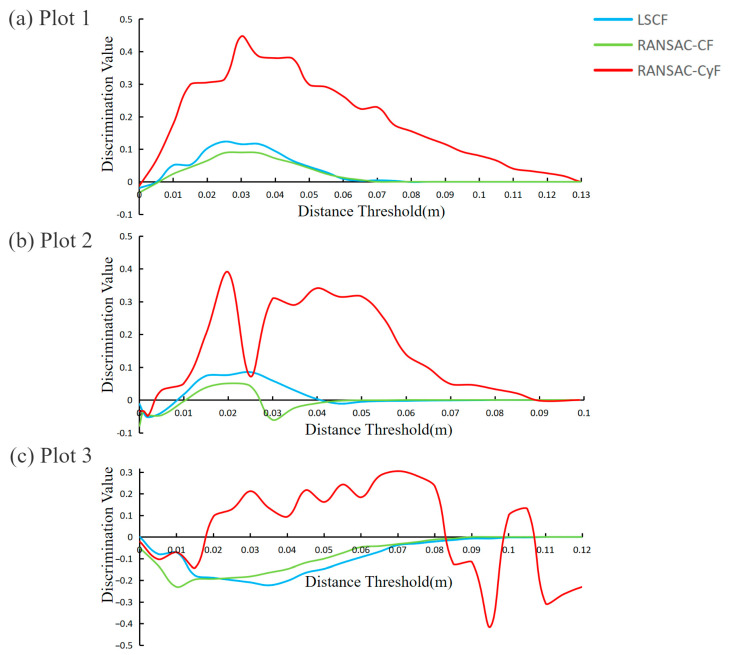
Distribution of discrimination values with varying distance thresholds for the three algorithms: (**a**) Plot 1; (**b**) Plot 2; (**c**) Plot 3.

As shown in Figure 10, in Plot 1, the effective distance threshold range for the LSCF and RANSAC-CF algorithms was approximately 0.005–0.07 m, which was much smaller than the effective range of 0.003–0.13 m for the RANSAC-CyF algorithm. In Plot 2, all three algorithms’ effective distance threshold ranges decreased. The LSCF and RANSAC-CF algorithms’ effective ranges decreased to 0.01–0.04 m and 0.01–0.025 m, respectively. However, the RANSAC-CyF algorithm was less affected, maintaining an effective range of 0.005–0.09 m. Regarding the numerical size of the discrimination value, the effective discrimination values of the RANSAC-CyF algorithm were significantly higher than those of the other two algorithms (*p* < 0.05). In Plot 3, the discrimination values of the LSCF and RANSAC-CF algorithms were less than or equal to 0 across all distance thresholds, while the effective range for the RANSAC-CyF algorithm was approximately 0.02–0.08 m.

### 3.4. Fitting Results for Tilted Trunks

Since the trees in the three plots are relatively upright, assessing the impact of trunk tilt on the algorithms’ fitting accuracy is difficult. Therefore, this experiment selected point clouds 18 and 28 from Plot 1 to represent complete and incomplete trunk point clouds, respectively. The point clouds were rotated around the center point towards the horizontal direction to simulate tilted trunks, and the changes in the inlier rates of the three algorithms were analyzed at different tilt angles.

**Figure 11:**
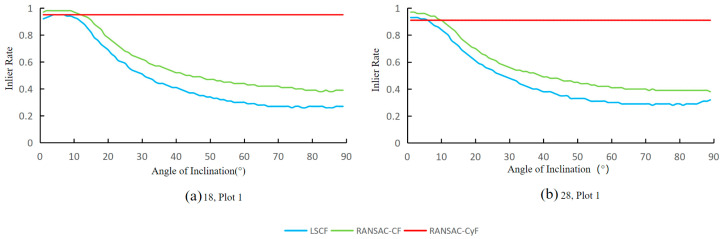
Changes in fitting inlier rates of the three algorithms at different tilt angles: (**a**) 18, Plot 1; (**b**) 28, Plot 1.

As shown in Figure 11, for point clouds 18 and 28, the fitting inlier rates of the LSCF and RANSAC-CF algorithms exhibited consistent patterns with varying tilt angles: for tilt angles less than 10 degrees, the inlier rates of LSCF and RANSAC-CF were both greater than 0.9. As the tilt angle increased, the inlier rates of LSCF and RANSAC-CF gradually decreased. At 30 degrees, the inlier rates of LSCF and RANSAC-CF dropped to approximately 0.5 and 0.6, respectively, and at 60 degrees, they further decreased to about 0.4 and 0.3. In contrast, the RANSAC-CyF algorithm showed stable inlier rates at all tilt angles, consistently greater than 0.9.

## 4. Discussion

**Figure 12:**
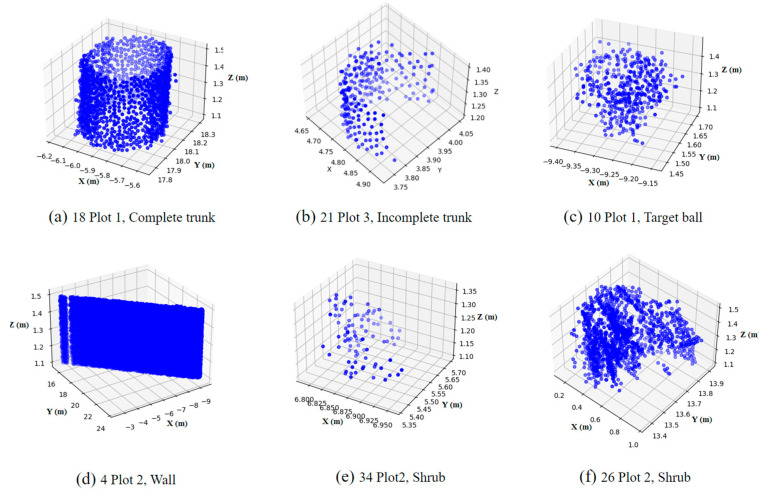
Plot point clouds: (**a**) 18 Plot 1, Complete trunk; (**b**) 21 Plot 3, Incomplete trunk; (**c**) 10 Plot 1, Target ball; (**d**) 4 Plot 2, Wall; (**e**) 34 Plot 2, Shrub; (**f**) 26 Plot 2, Shrub.

The experimental data include complete and incomplete trunk point clouds and non-trunk point clouds such as target ball point clouds, wall point clouds, and shrub point clouds (Figure 12).

**Figure 13:**
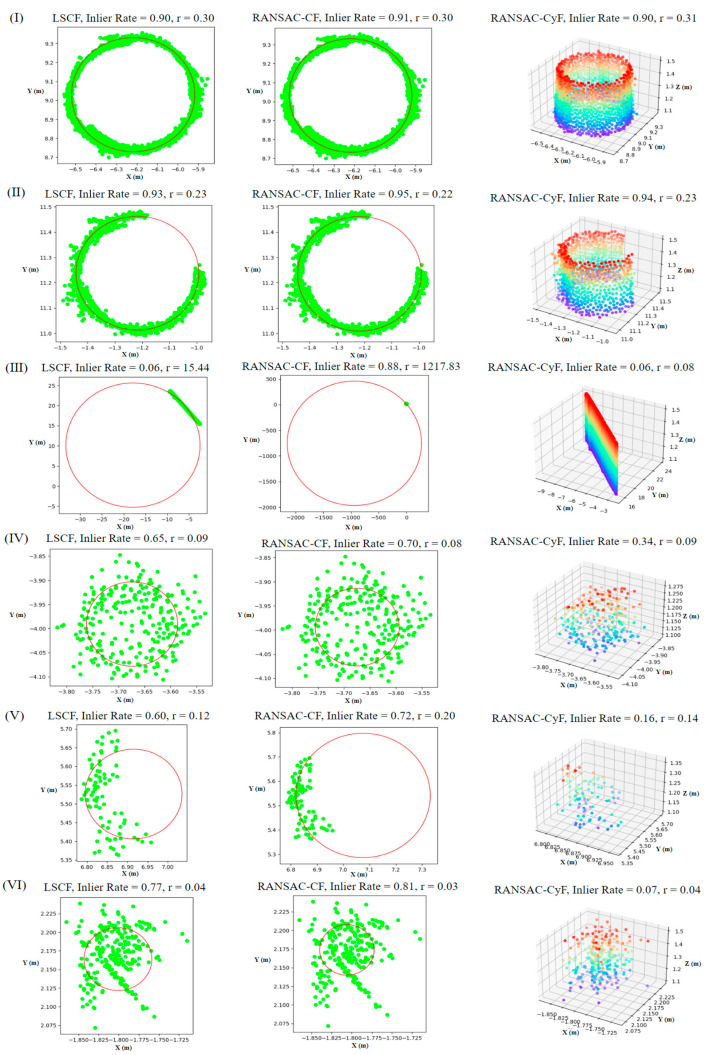
Comparison of fitting results for selected point clouds by the three algorithms: (**I**) complete trunk point cloud (16 Plot1), (**II**) incomplete trunk point cloud (28 Plot 1), (**III**) wall point cloud (4 Plot 2), (**IV**) target ball point cloud (20 Plot 1), and (**V**,**VI**) shrub point clouds (34, 18 Plot 2).

For trunk point clouds, regardless of whether the trunk is complete, the LSCF, RANSAC-CF, and RANSAC-CyF algorithms all demonstrated excellent fitting results (Figure 8 and Figure 13I,II). In Plot 1, the average inlier rates of the three algorithms exceeded 0.9, while in Plot 2, they reached 0.92, 0.91, and 0.71, respectively. In Plot 3, all three algorithms achieved average inlier rates above 0.85.

However, for non-trunk point clouds, the fitting results of the three algorithms showed significant differences. In Plot 2, the inlier rates of the LSCF and RANSAC-CF algorithms were unstable for certain point clouds (e.g., 6, 18, 28, 35), with some inlier rates as high as 0.8 (Figure 8). This was mainly due to the circular or semi-circular planar projections of non-trunk point clouds, such as target ball point clouds (Figure 13IV) and certain shrub point clouds (Figure 13VI), causing the inlier rates of the LSCF and RANSAC-CF algorithms to exceed 0.6, with some shrub point clouds even exceeding 0.8 (Figure 13VI). In contrast, the RANSAC-CyF algorithm, based on 3D RANSAC cylinder fitting, effectively handled these cases as non-trunk point clouds like shrubs, walls, and spherical target balls that do not conform to the cylinder model. As a result, the inlier rates of the RANSAC-CyF algorithm for non-trunk point clouds remained consistently low and stable, with averages of 0.31 in Plot 1 and 0.08 in Plot 2.

In Plot 3, the average inlier rates of the LSCF and RANSAC-CF algorithms for non-trunk point clouds were as high as 0.84 and 0.88, rendering them nearly incapable of distinguishing trunks. This is because the trees in Plot 3 are smaller, with an average DBH of 0.248 m. The point cloud stitching precision is 0.03 m, and their shapes are less cylindrical compared to the Royal Palm trees in Plots 1 and 2 (0.491 m). These factors together caused the 2D planar algorithms to fail in accurately distinguishing trunks. Although the RANSAC-CyF algorithm was also affected, its inlier rates for non-trunk point clouds remained below 0.5, maintaining relatively good discrimination capability.

The inlier rate results of trunk and non-trunk point clouds indicate that high inlier rates alone cannot accurately identify trees. An effective individual tree detection algorithm should be capable of efficiently identifying individual trees and excluding non-trunk point clouds. Therefore, the difference between the minimum inlier rate for trunk point clouds and the maximum inlier rate for non-trunk point clouds, referred to as the discrimination value, can better distinguish trunk and non-trunk point clouds. The RANSAC-CyF algorithm achieved discrimination values of 0.447 and 0.390 in Plots 1 and 2, respectively, significantly higher than those of the LSCF and RANSAC-CF algorithms. Even in the more challenging Plot 3, it achieved a discrimination value of 0.305, demonstrating better adaptability to point cloud data precision, tree species, and trunk morphology. Benefiting from its larger discrimination value, the RANSAC-CyF algorithm allows for easier and more accurate inlier rate threshold settings with fewer samples, showcasing superior individual tree detection performance and practical applicability.

However, accurately setting the optimal distance threshold in practical applications is challenging [17,20]. Therefore, an excellent algorithm should exhibit robustness in selecting the distance threshold and have a considerable discrimination value. The robustness analysis results showed that the effective distance threshold range and the corresponding discrimination values of the RANSAC-CyF algorithm in all three plots were much more extensive than those of the other two algorithms, demonstrating the superior robustness and adaptability of the RANSAC-CyF algorithm to complex environments.

**Figure 14:**
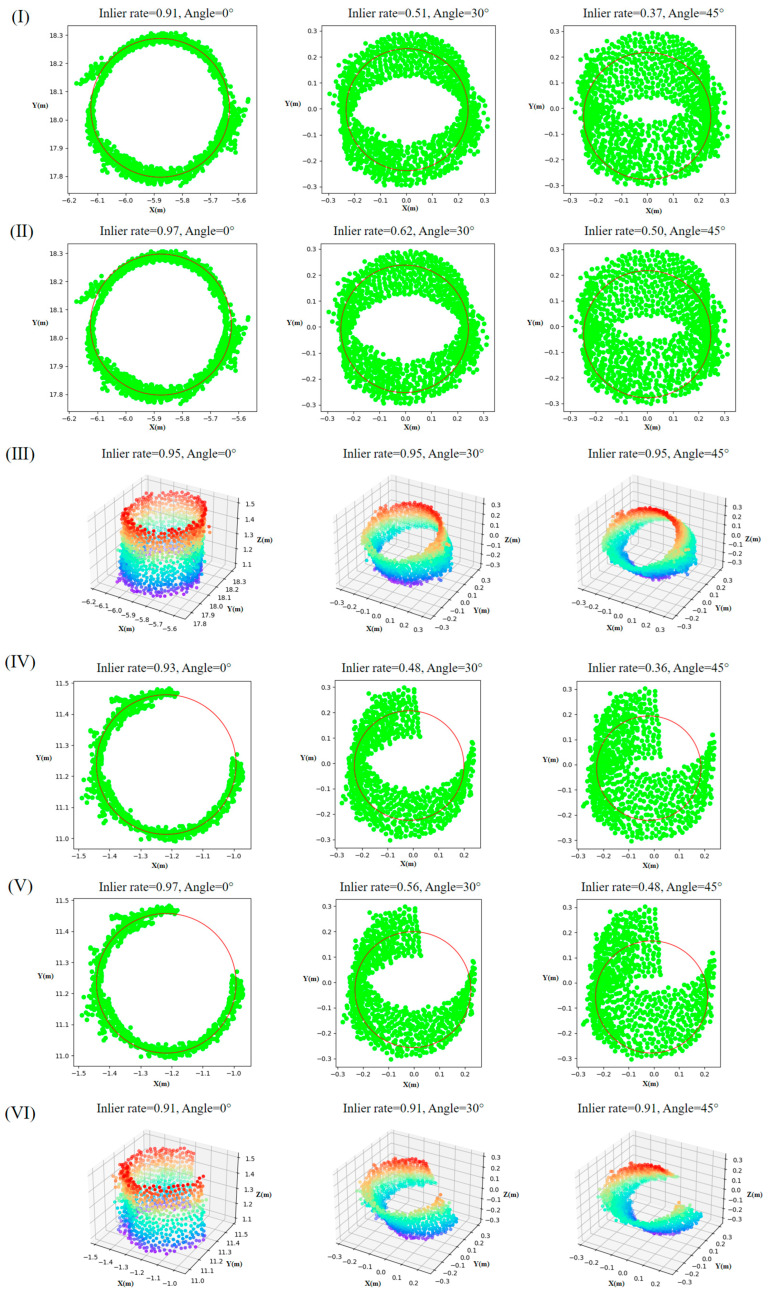
Comparison of fitting results for tilted trunk point clouds by the three algorithms: (**I**–**III**): LSCF, RANSAC-CF, RANSAC-CyF) complete trunk point cloud (18, Plot 1), (**IV**–**VI**): LSCF, RANSAC-CF, RANSAC-CyF) incomplete trunk point cloud (28, Plot 1).

Additionally, in the analysis of tilted trunks, as the tilt angle increases, the point cloud planar projection of the LSCF and RANSAC-CF algorithms gradually changes from a circle to an ellipse (Figure 11, Figure 12 and Figure 14), reducing the inlier rate [7]. In contrast, the 3D RANSAC-CyF algorithm’s inlier rate is unaffected by trunk tilt, maintaining a consistent inlier rate at different tilt angles [13]. Therefore, the RANSAC-CyF algorithm effectively addresses the issue of reduced inlier rates in LSCF and RANSAC-CF algorithms caused by tilted trees.

The experimental results from Plot 3 demonstrated that the proposed algorithm is applicable not only to Royal Palm trees but also to small trees with shapes that are not fully cylindrical. However, for tree species with shapes that significantly differ from a cylindrical model (e.g., trees with heavily branched or twisted trunks), the algorithm may face a decline in detection accuracy. This limitation is primarily due to the current algorithm’s assumption that trunks follow a cylindrical model, which may fail to accurately distinguish trunks from non-trunk point clouds in complex point cloud data. In the future, the algorithm could be improved by optimizing the fitting model (e.g., supporting elliptical or complex surface fitting) or incorporating machine learning methods to enhance adaptability to irregular trunk shapes.

## 5. Conclusions

By comparing the individual tree detection analyses of the RANSAC-CyF algorithm, LSCF algorithm, and RANSAC-CF algorithm on the data from three plots, the following conclusions are drawn:

(1) The RANSAC-CyF algorithm’s ability to detect individual trees is superior to that of the LSCF and RANSAC-CF algorithms. In Plots 1, 2, and 3, the discrimination values of the RANSAC-CyF algorithm were 0.447, 0.39, and 0.305, respectively, significantly higher than those of the LSCF and RANSAC-CF algorithms (both less than 0.15).
(2) The RANSAC-CyF algorithm exhibited an excellent success rate in individual tree detection. In Plot 1, only two samples were needed to achieve a 100% perfect individual tree detection success rate, and in Plot 2, the success rate reached 91%. Even in the more challenging Plot 3, only 10 samples were required to achieve an 80% success rate, making the algorithm more accurate and convenient in practical applications.
(3) The RANSAC-CyF algorithm is robust in setting the distance threshold. The effective distance threshold ranges for the RANSAC-CyF algorithm in Plot 1, 2, and 3 were 0.003–0.13 m, 0.005–0.09 m, and 0.02–0.08 m, respectively, more than twice those of the LSCF and RANSAC-CF algorithms.
(4) The RANSAC-CyF algorithm’s inlier rate for fitting was not affected by trunk tilt, effectively addressing the issue faced by the LSCF and RANSAC-CF algorithms where the inlier rate decreases with tilted trees.

Overall, the RANSAC-CyF algorithm outperformed the LSCF and RANSAC-CF algorithms in terms of individual tree detection accuracy, inlier rate threshold range, stability, and robustness. Therefore, the individual tree detection algorithm based on RANSAC cylinder fitting solves the problems of information loss and inaccurate detection results caused by trunk tilt in traditional 2D circle fitting algorithms. Through experiments, the RANSAC-CyF algorithm has demonstrated high accuracy in individual tree detection and strong adaptability in complex forest scenes, significantly improving the efficiency and accuracy of individual tree detection in forestry surveys and ecological research.

## Figures and Tables

**Table 1:** Comparison of total clusters and trunk point cloud clusters in the plots.

Plot Number	Total Number of Clusters	Trunk Point Cloud Clusters
Plot 1	30	27
Plot 2	53	25
Plot 3	26	23

**Table 2:** Optimal discrimination values of the three algorithms. (Values are not reported for cases where the discrimination value is ≤0).

Plot Number	Method	Inlier Rate Range	Discrimination Value	Distance Threshold (m)
1	LSCF	(0.709, 0.832)	0.123	0.025
	RANSAC-CF	(0.768, 0.850)	0.082	0.03
	RANSAC-CyF	(0.351, 0.798)	0.447	0.03
2	LSCF	(0.797, 0.882)	0.085	0.025
	RANSAC-CF	(0.805, 0.853)	0.048	0.025
	RANSAC-CyF	(0.177, 0.567)	0.390	0.02
3	LSCF	--	--	--
	RANSAC-CF	--	--	--
	RANSAC-CyF	(0.489, 0.794)	0.305	0.07

## Data Availability

The point cloud data in this study can be obtained from the corresponding author.

## References

[1] Li Z., Liu Q., Pang Y. (2016). Research Progress on Forest Parameter Inversion Using LiDAR. J. Remote Sens..

[2] Akerblom M., Kaitaniemi P. (2021). Terrestrial Laser Scanning: A New Standard of Forest Measuring and Modelling?. Ann. Bot..

[3] Hui Z., Jin S., Li D., Ziggah Y.Y., Liu B. (2021). Individual Tree Extraction from Terrestrial LiDAR Point Clouds Based on Transfer Learning and Gaussian Mixture Model Separation. Remote Sens..

[4] Reddy R.S., Jha C.S., Rajan K.S. (2018). Automatic Tree Identification and Diameter Estimation Using Single Scan Terrestrial Laser Scanner Data in Central Indian Forests. J. Indian Soc. Remote.

[5] Olofsson K., Holmgren J., Olsson H. (2014). Tree Stem and Height Measurements using Terrestrial Laser Scanning and the RANSAC Algorithm. Remote Sens..

[6] Ning X., Ma Y., Hou Y., Lv Z., Jin H., Wang Z., Wang Y. (2023). Trunk-Constrained and Tree Structure Analysis Method for Individual Tree Extraction from Scanned Outdoor Scenes. Remote Sens..

[7] Bienert A., Georgi L., Kunz M., von Oheimb G., Maas H. (2021). Automatic extraction and measurement of individual trees from mobile laser scanning point clouds of forests. Ann. Bot..

[8] Maas H.G., Bienert A., Scheller S., Keane E. (2008). Automatic Forest Inventory Parameter Determination from Terrestrial Laser Scanner Data. Int. J. Remote Sens..

[9] Singh A., Kushwaha S.K.P., Nandy S., Padalia H. (2022). An Approach for Tree Volume Estimation Using RANSAC and RHT Algorithms from TLS Dataset. Appl. Geomat..

[10] Calders K., Newnham G., Burt A., Murphy S., Raumonen P., Herold M., Culvenor D., Avitabile V., Disney M., Armston J. (2015). Nondestructive Estimates of Above-Ground Biomass Using Terrestrial Laser Scanning. Methods Ecol. Evol..

[11] Liang X., Hyyppä J. (2013). Automatic Stem Mapping by Merging Several Terrestrial Laser Scans at the Feature and Decision Levels. Sensors.

[12] Feng B., Nie S., Wang C., Xi X., Wang J., Zhou G., Wang H. (2022). Exploring the Potential of UAV LiDAR Data for Trunk Point Extraction and Direct DBH Measurement. Remote Sens..

[13] Horiuchi E. (2013). Automatic Detection of Tree-Trunks by Fitting Cylinders to 3D Point Clouds. J. Robot. Soc. Jpn..

[14] Yang J., Yuan W., Lu H., Liu Y., Wang Y., Sun L., Li S., Li H. (2024). Assessing the Performance of Handheld Laser Scanning for Individual Tree Mapping in an Urban Area. Forests.

[15] Yang Q., Chen H., Ma Z., Xu Y., Tang R., Sun J. (2021). Predicting the Perceptual Quality of Point Cloud: A 3D-to-2D Projection-Based Exploration. IEEE Trans. Multimed..

[16] Shuang F., Li P., Li Y., Zhang Z., Li X. (2022). MSIDA-Net: Point Cloud Semantic Segmentation via Multi-Spatial Information and Dual Adaptive Blocks. Remote Sens..

[17] Sheng Y., Zhao Q., Wang X., Liu Y., Yin X. (2024). Tree Diameter at Breast Height Extraction Based on Mobile Laser Scanning Point Cloud. Forests.

[18] Liu Q., Ma W., Zhang J., Liu Y., Xu D., Wang J. (2021). Point-Cloud Segmentation of Individual Trees in Complex Natural Forest Scenes Based on a Trunk-Growth Method. J. For. Res..

[19] Li L., Yang F., Zhu H., Li D., Li Y., Tang L. (2017). An Improved RANSAC for 3D Point Cloud Plane Segmentation Based on Normal Distribution Transformation Cells. Remote Sens..

[20] Zhang T., Wei L., Tang H., Wang L., Yuan M., Niu X. (2023). SE-LIO: Semantics-Enhanced Solid-State-LiDAR-Inertial Odometry for Tree-Rich Environments. arXiv.

[21] Chung K., Tseng Y., Chen H. (2022). A Novel and Effective Cooperative RANSAC Image Matching Method Using Geometry Histogram-Based Constructed Reduced Correspondence Set. Remote Sens..

[22] Trochta J., Krucek M., Vrska T., Kral K. (2017). 3D Forest: An Application for Descriptions of Three-Dimensional Forest Structures Using Terrestrial LiDAR. PLoS ONE.

[23] Eysn L., Hollaus M., Lindberg E., Berger F., Monnet J., Dalponte M., Kobal M., Pellegrini M., Lingua E., Mongus D. (2015). A Benchmark of LiDAR-Based Single Tree Detection Methods Using Heterogeneous Forest Data from the Alpine Space. Forests.

[24] Gupta S., Weinacker H., Koch B. (2010). Comparative Analysis of Clustering-Based Approaches for 3D Single Tree Detection Using Airborne Fullwave LiDAR Data. Remote Sens..

[25] Holcomb A., Tong L., Keshav S. (2023). Robust Single-Image Tree Diameter Estimation with Mobile Phones. Remote Sens..

[26] Yubo L., Hongyu H., Liyu T., Chongcheng C., Hao Z. (2019). Tree Height and Diameter Extraction with 3D Reconstruction in a Forest based on TLS. Remote Sens. Technol. Appl..

[27] Muumbe T.P., Baade J., Singh J., Schmullius C., Thau C. (2021). Terrestrial Laser Scanning for Vegetation Analyses with a Special Focus on Savannas. Remote Sens..

[28] Schrum P.T., Jameson C.D., Tateosian L.G., Blank G.B., Wegmann K.W., Nelson S.A.C. (2023). Curvature Weighted Decimation: A Novel, Curvature-Based Approach to Improved LiDAR Point Decimation of Terrain Surfaces. Geomatics.

[29] Tran T., Cao V., Laurendeau D. (2015). Extraction of cylinders and estimation of their parameters from point clouds. Comput. Graph..

[30] Tarsha Kurdi F., Gharineiat Z., Lewandowicz E., Shan J. (2024). Modeling the Geometry of Tree Trunks Using LiDAR Data. Forests.

[31] Young M., Pretty C., Agostinho S., Green R., Chen X. (2019). Loss of Significance and Its Effect on Point Normal Orientation and Cloud Registration. Remote Sens..

[32] Skrodzki M. (2019). The k-d tree data structure and a proof for neighborhood computation in expected logarithmic time. arXiv.

[33] Rusu R.B., Cousins S. (2011). In 3D is here: Point Cloud Library (PCL). Proceedings of the 2011 IEEE International Conference on Robotics and Automation.

[34] Raguram R., Chum O., Pollefeys M., Matas J., Frahm J. (2013). USAC: A Universal Framework for Random Sample Consensus. Ieee Trans. Pattern Anal..

[35] Sakib M.N., Rahman M.A. (2018). Traffic Bottleneck Reconstruction LIDAR Orthoimages: A RANSAC Algorithm Feature Extraction. Recent Trends in Data Science and Soft Computing.

[36] Fotouhi M., Hekmatian H., Kashani-Nezhad M.A., Kasaei S. (2019). SC-RANSAC: Spatial consistency on RANSAC. Multimed. Tools Appl..

